# Employments for Women

**Published:** 1891-08-22

**Authors:** 


### Employments for Women.

### III.—Gardening.

Why should we accept for gospel the statement made, or implied, in the old couplet which asks us, "When Adam dolve and Eve span, Who was then the gentleman?" For our own part we believe that Eve *did* delve, and that she did many a delicate little job besides, which Adam was quick to see that her fingers were more suited for than his. But be this as it may, we see no reason at all why her daughters should not engage in this pursuit, and cut a very good figure in it too, if they are only steady and persevering. Sad to relate, we have heard rumours that some young ladies are apt to be impatient about their horticultural work—not, perhaps, going quite so far as to pull up their plants by the roots to see if there is no sign of growth, but doing what is pretty nearly an equivalent to it; and there *is* a story—a well-accredited one, we believe—that a lady pupil at a horticultural college threw up her work for good and all on the ground that certain steels upon which she had reckoned for support had the objectionable habit of snapping whenever she stooped! Well, but there are girls and girls—those who have been always used to "frivol" away their time and talents, and hardly know how to do anything else, and those who are really resolved to fit themselves for useful work. These latter, we are sure, will find gardening a very suitable as well as healthful pursuit; and there seems no reason why it should not also prove a paying one.

We can hardly think that schemes are likely to answer which propose to make a lady with a little capital, but no experience, into a market gardener straight away. In this, as in almost everything else, some preliminary training must surely be needed, and we would strongly advise every woman who thinks of taking up this occupation to go in for a good course of training before making a start for herself. The importance of this has been recognised in various quarters. Alexandra College, Dublin, has introduced horticulture for women, and Holloway College intends to, if it has not done so already; while Miss Buss, of the North London Collegiate School, takes the warmest interest in the matter, her name appearing as one of the Patronesses of the lately formed Ladies' Branch of the Swanley Horticultural College.

We do not think that a girl wishing to learn horticulture could possibly do better than enter upon a year's, or possibly two years', course of training at this college. Right in the heart of the fruit-growing district of Kent, yet within sixteen miles of London, it stands, a fine avenue of trees leading up to a bright looking, modernised old house, with fine lecture-rooms (one of them is the saloon of the "Bessemer," by the way, and a very handsome room it makes), a chemical laboratory, and all conveniences for the students; while round and about are peach houses, vineries, tomato and cucumber houses (these in great quantity), mushroom houses, poultry run, dairy, apiary, &c., &c. On the day we visited it, too, there was a fine show of geraniums and roses for the London market, to which are sent consignments of fruit, flowers, and vegetables every other day. Then there are the strawberry gardens, and the orchards, where between the gnarled trunks of the apple trees flourish the gooseberry and the currant, the grounds altogether being over forty-three acres in extent.

No sooner do you leave the station (a mile and a-half from the college), than in summer time, at any rate, you seem to think, to smell, to breathe, to live fruit. You may bet ten to one that the sturdy country people whom you overtake in the lanes will be babbling of strawberries, or fruit of some kind—what else have they to think and talk about? And the air is so pure, the prospect so fair, that you feel that if only Time would turn backward, and give you the choice of a life's occupation over again, you would certainly choose gardening, and enter yourself in the twinkling of an eye at this delightful college.

But the lady students do not live at the college—no, no. Adam meets Eve in the lecture-hall, and perhaps wanders about the grounds, and in and out of the glass-houses, with her, at the tail of the "Practical Instructor"; but there their intercourse ceases, and Eve goes back to a little paradise of her own—a pretty and delightfully fresh and clean-looking house within five minutes' walk of the college. Here there is accommodation for ten or twelve young ladies, the head of the house being a widow lady, who made a very pleasing impression upon us, and will, we feel sure, win the respect and affection of her girl-boarders. One lady has been a student at the college for some little time, three or four others are joining this term, and we can hardly doubt that others will quickly follow.

The fees for a lady pupil, including everything but laundress, books, and fire in bed-room, are £70, or if a separate room is required, £80 per annum. Students are expected to devote five hours a-day to practical, as well as three to theoretic work, and should provide themselves with strong, plainly-made dresses, stout boots, and large bibbed aprons. The holidays consist of a month at Christmas, and another at Midsummer, these being the times at which horticultural work is least pressing. We may mention that arrangements can be made for ladies wishing to pay a short temporary visit; but anyone wishing for full particulars will do well to write to the Hon. Secretary, Mrs. Chamberlain, 55, Drayton Gardens, S.W., or to the Lady Superintendent, Mrs. Watson, 2, Hextable Villas, Swanley, Kent.

As to the use which may eventually be made of such training, we see no reason why a lady should not take a post as upper gardener in a large gentleman's place, or as "gardening lady-help" in a smaller; but her best chance will probably be to garden for herself, turning to good account the garden which she already has, or taking on one for the purpose, and disposing of its produce either to the trade, or privately to her friends. Excellent little businesses may be made in this way, one customer recommending another, and so on; and as the lady gardener's motto must be "early to bed and early to rise," we may hope that she will exemplify the truth of the proverb, and speedily become "healthy and wealthy and wise."

